# Identification of Functional Immune Biomarkers in Breast Cancer Patients

**DOI:** 10.3390/ijms252212309

**Published:** 2024-11-16

**Authors:** Roshanak Derakhshandeh, Yuyi Zhu, Junxin Li, Danubia Hester, Rania Younis, Rima Koka, Laundette P. Jones, Wenji Sun, Olga Goloubeva, Katherine Tkaczuk, Joshua Bates, Jocelyn Reader, Tonya J. Webb

**Affiliations:** 1 Department of Microbiology and Immunology, University of Maryland School of Medicine, Baltimore, MD 21201, USA; roshanak.derakhshandeh@gunet.georgetown.edu (R.D.); yuyizhu1@som.umaryland.edu (Y.Z.); celine.junxin@gmail.com (J.L.); danubia.hester@usoncology.com (D.H.); wsun@som.umaryland.edu (W.S.); joshua.bates@umaryland.edu (J.B.); 2 Department of Oncology and Diagnostic Sciences, University of Maryland School of Dentistry, Baltimore, MD 21201, USA; rania.younis@gmail.com; 3 Marlene and Stewart Greenebaum Comprehensive Cancer Center, University of Maryland, Baltimore, Baltimore, MD 21201, USA; mkoka@som.umaryland.edu (R.K.); lpjones@som.umaryland.edu (L.P.J.); ogoloubeva@som.umaryland.edu (O.G.); ktkaczuk@umm.edu (K.T.); jreader@umes.edu (J.R.); 4 Department of Pathology, University of Maryland School of Medicine, Baltimore, MD 21201, USA; 5 Department of Epidemiology and Public Health, University of Maryland School of Medicine, Baltimore, MD 21201, USA; 6 Department of Pharmaceutical Sciences, University of Maryland Eastern Shore, Princess Anne, MD 21853, USA; 7 Department of Obstetrics, Gynecology and Reproductive Sciences, University of Maryland School of Medicine, Baltimore, MD 21201, USA

**Keywords:** breast cancer, immune response, NKT cells, lipids, statins, CD1d, BRCA

## Abstract

Cancer immunotherapy has emerged as an effective, personalized treatment for certain patients, particularly for those with hematological malignancies. However, its efficacy in breast cancer has been marginal—perhaps due to cold, immune-excluded, or immune-desert tumors. Natural killer T (NKT) cells play a critical role in cancer immune surveillance and are reduced in cancer patients. Thus, we hypothesized that NKT cells could serve as a surrogate marker for immune function. In order to assess which breast cancer patients would likely benefit from immune cell-based therapies, we have developed a quantitative method to rapidly assess NKT function using stimulation with artificial antigen presenting cells followed by quantitative real-time PCR for IFN-γ. We observed a significant reduction in the percentage of circulating NKT cells in breast cancer patients, compared to healthy donors; however, the majority of patients had functional NKT cells. When we compared BC patients with highly functional NKT cells, as indicated by high IFN-γ induction, to those with little to no induction, following stimulation of NKT cells, there was no significant difference in NKT cell number between the groups, suggesting functional loss has more impact than physical loss of this subpopulation of T cells. In addition, we assessed the percentage of tumor-infiltrating lymphocytes and PD-L1 expression within the tumor microenvironment in the low and high responders. Further characterization of immune gene signatures in these groups identified a concomitant decrease in the induction of TNFα, LAG3, and LIGHT in the low responders. We next investigated the mechanisms by which breast cancers suppress NKT-mediated anti-tumor immune responses. We found that breast cancers secrete immunosuppressive lipids, and treatment with commonly prescribed medications that modulate lipid metabolism, can reduce tumor growth and restore NKT cell responses.

## 1. Introduction

Gene expression signatures have been used to classify breast tumors into subtypes showing distinct molecular expression profiles associated with specific clinical characteristics [1,2,3,4,5,6]. While surgery, radiotherapy, chemotherapy, and targeted therapies have drastically improved 5-year survival rate of patients, significant efforts have been invested into harnessing the therapeutic potential of the immune system for the treatment of cancer [7,8,9]. The main goal of immunotherapy is to use the body’s own immune system to eradicate cancer cells. Despite notable successes, responses to immunotherapy interventions only occur in a minority of patients [10]. Attempts are being made to improve the activity of immunotherapies with novel combinational strategies and the identification of reliable biomarkers [11]. The results from several recent clinical trials have highlighted the potential for combination therapies based on the patient’s tumor gene expression pattern [12,13,14]. Therefore, there is a need of reliable ways to assess patient immune profiles in order to guide personalized strategies such as immunotherapy.

Immunotherapy is one strategy used to enhance immune responses in cancer patients. Many groups have used a diverse array of methods involving genetic modification or viral transduction to generate large numbers of tumor-specific T cells for adoptive immunotherapy. An alternate cellular strategy in development utilizes natural killer (NK) cells, a component of the innate immune system. These NK cells directly lyse cancer cells, and produce cytokines and chemokines that can activate other components of the immune system. In these studies, we sought to examine a third type of cellular therapy. Natural killer T cells (NKT cells) were discovered in the late 1980s and were named based on the fact that they display features of both NK cells and classical T cells. Importantly, NKT cells can active both NK cells and classical T cells. Their dual function is ideal for cancer treatment because NKT cell-based therapy offers the possibility of inducing an initial cytotoxic tumor response and activating the adaptive immune system to produce tumor-directed cytotoxic T cells (CTL) with long-lived memory. Clinical trials using NKT cell-based immunotherapy have been conducted in patients with non-small cell lung cancers [15,16], and solid tumors [17]. However, studies from our group and others have reported a significant reduction in NKT cells in cancer patients [18,19]. Moreover, several groups have shown a correlation between the number of NKT cells and survival rate in colon cancer [20] and head and neck squamous cell carcinoma [21]. They observed poor prognosis in patients with a decreased number of NKT cells [22]. A recent report on head and neck cancer suggested an association between an IFN-γ signature and clinical response [23]. Collectively, these studies suggest that NKT cell-based therapies have potential in patients with functional NKT cells and further implicate a role for NKT cells as a potential diagnostic biomarker. Thus, the primary aim of our study was to assess the efficacy of utilizing NKT cell function as an immune biomarker in breast cancer. We used artificial antigen-presenting cells (aAPC) to stimulate peripheral blood NKT cells in healthy donors and cancer patients. We measured IFN-γ induction and identified other immune-related genes that were induced in breast cancer patients. Our findings have a potential to introduce a screening biomarker for immune response, which can help to personalize immunotherapy in breast cancer patients.

## 2. Results

### 2.1. Patient Demographics

Our lab has generated a platform based on stimulating cells with artificial antigen-presenting cells (aAPC) that can be used to assess NKT function in peripheral blood mononuclear cells (PBMC) of healthy donors. In this study, we sought to determine if this method could measure baseline immune function in breast cancer patients. PBMC were collected from 30 newly diagnosed breast cancer (BC) patients (Table 1). At diagnosis, 50% of BC patients were older than 50 years of age. The median age was 51.5 ± 12.3 years old (range 20–89) and our study included a population that was 43.3% White, 46.6% African American, and 10% Asian. Most BC patients had ductal carcinoma (80%). The majority of tumors expressed estrogen receptor (ER), progesterone receptor (PR), or both; however, 26.6% of BC patients were classified as a triple-negative breast cancer (TNBC). There were similar numbers of patients in each tumor grade and stage category. We observed a slight reduction in number and function of NKT cells above 50 years of age, but this difference was not statistically significant. While higher NKT function was detected in African Americans, compared to other ethnicities, the difference was not significant.

### 2.2. aAPC–qPCR Can Be Used to Assess NKT Cell Activation

**Figure 1:**
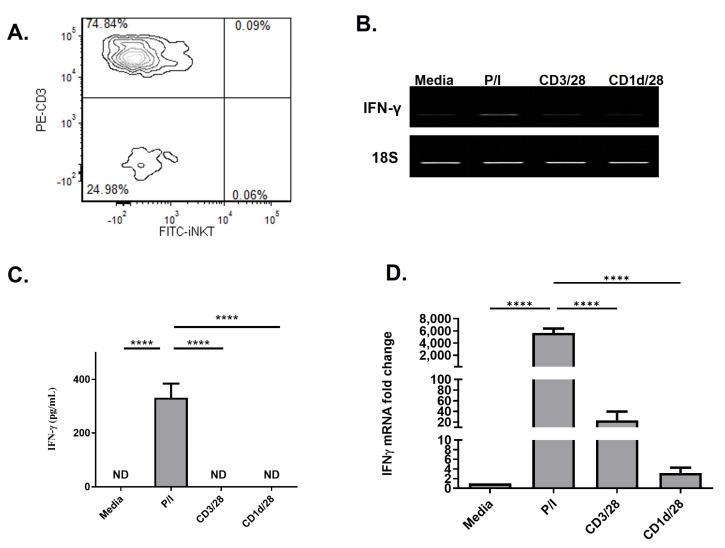
NKT cell function in breast cancer patients can be assessed using CD1d-based aAPC in combination with qPCR. (**A**) Flow cytometric analysis of circulating NKT cells from a breast cancer (BC) patient. PBMC from breast cancer patients were incubated for 4 h with media, CD1d-aAPC, anti-CD3/CD28 microbeads, or PMA/ionomycin (P/I). NKT cell activation was assessed by measuring IFN-γ mRNA levels by (**B**) RT-PCR, (**C**) ELISA, and (**D**) qPCR. Representative data are shown from one patient; however, this characterization was performed on each BC patient in this study (n = 30). A one-way ANOVA was performed with a Tukey multiple comparison test with *p* ≤ 0.05 considered significant. **** *p* ≤ 0.0001.

As shown in Figure 1A, the frequency of NKT cells in the peripheral blood of BC patients is relatively low. Due to the low percentage of circulating NKT cells, we next sought to determine if stimulation with aAPC followed by qPCR could be used to measure NKT cell function in BC patients. PBMCs were stimulated with α-GalCer loaded aAPC and IFN-γ induction was measured using RT-PCR, ELISA, and qPCR. We found that conventional methods such as RT-PCR (Figure 1B) and ELISA (Figure 1C) are not sensitive enough to assess NKT cell activation following a four-hour activation period; however, qPCR can detect the induction of NKT cell activation in cancer patients. Thus, we have developed a robust cell-based molecular diagnostic for quantitation of a patient’s NKT cell activity by monitoring IFN-γ induction utilizing qPCR post activation with CD1d-based aAPC.

### 2.3. aAPC–qPCR Is a Valid Platform in Healthy Donors as Well as Breast Cancer Patients

**Figure 2:**
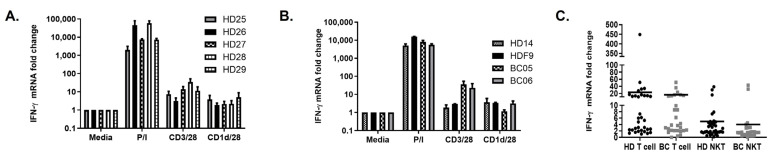
Validation of aAPC–qPCR platform in healthy donors and breast cancer patients. The aAPC–qPCR platform can be used to detect NKT/T cell function in cells from (**A**) fresh blood, (**B**) frozen blood (HDF9), and breast cancer patient samples (BC05 and BC06). A two-way ANOVA was performed with a Tukey multiple comparison test with *p* ≤ 0.05 considered significant. For all comparisons, only comparisons relative to P/I controls were significant (*p* ≤ 0.05). (**C**) Peripheral blood mononuclear cells (PBMC) were stimulated with anti-CD3/CD28 microbeads to stimulate T cells or CD1d-Ig/αCD28 aAPC loaded with α-GalCer to stimulate NKT cells for 4 h. RNA was extracted, and qPCR was performed to assess IFN-γ and 18S. Fold induction was calculated relative to the control (cells stimulated with empty beads). The results shown in A and C are based on 18 HD and 30 BC patients. Fresh and frozen samples were compared from five healthy donors. (P/I indicates PMA/ionomycin).

qPCR was used to assess IFN-γ induction following stimulation of all PBMCs used in this study, including eighteen female healthy donors (HD), five frozen HD samples, and thirty female BC patients. As shown in Figure 2A, we were able to detect T cell and NKT cell activation in the majority of HD. Furthermore, we compared the result of fresh HD PBMCs with frozen HD PBMCs and found no significant difference between fresh and frozen PBMCs (Figure 2B). Although the percentage of circulating NKT cells was significantly reduced in BC patients (% NKT = 0.044 ± 0.047) compared to HD (% NKT = 0.22 ± 0.54), NKT cell function was detectable in 88% of female HD (n = 16/18) and 73% of BC patients (n = 22/30). In addition, it was found that the majority of HD and BC patients had functional T cells (88% HD; 90% BC patients, Figure 2C).

**Figure 3:**
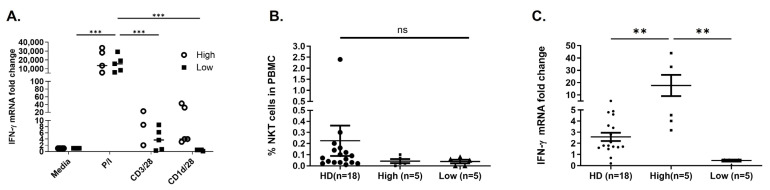
NKT cell number does not correlate with function in breast cancer patients. (**A**) aAPC–qPCR analysis of HD and BC patients with high NKT cell responses compared to those that were low responders based on IFN-γ induction. A two-way ANOVA was performed with a Tukey multiple comparison test was performed with *p* ≤ 0.05 considered significant. Only the comparisons relative to P/I were significant (*p* ≤ 0.001). (**B**) Flow cytometry analysis and (**C**) qPCR analysis of circulating NKT cells in HD and BC patients with high and low NKT cell function, as indicated by IFN-γ induction. A one-way ANOVA was performed with a Tukey multiple comparison test with *p* ≤ 0.05 considered significant. ns = not significant, ** *p* ≤ 0.01, *** *p* ≤ 0.001.

While the majority of breast cancer patients have functional NKT cells (assessed as IFN-γ fold induction > 1.5 fold higher than baseline), there was a cohort that did not have detectable NKT cell function. To identify the mechanisms accounting for the differences in responses, we compared five BC high responders (high IFN-γ induction) to five low responders (Table 2). As shown in Figure 3A, responses to the positive control (PMA/ionomycin) were similar in both groups. However, there is a striking reduction in NKT and T cell responses in the low responder group. When we examined the NKT population by flow cytometry, the percentage was reduced compared to HD, but no significant differences were detected in the percentage of circulating NKT cells in these two groups (Figure 3B). In contrast, there was a significant difference in NKT responses, as measured by IFN-γ induction. Thus, our data suggest that there is no correlation between the number and function of NKT cells in the peripheral blood of BC patients (Figure 3C).

### 2.4. Gene Expression Profiling Can Predict Candidates for NKT Cell-Based Immunotherapy

**Figure 4:**
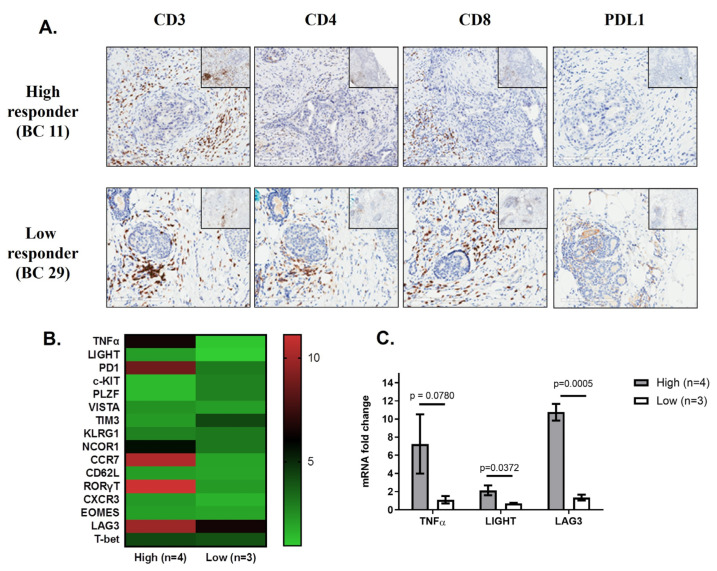
Gene profiles comparing breast cancer patients with high and low NKT cell function. (**A**) Immunohistochemical staining of CD3, CD4, CD8, and programmed death-ligand 1 (PD-L1) to characterize tumor-infiltrating lymphocytes. Representative photographs are shown from breast cancer microenvironment in high responder (upper panel) versus low responder (lower panel). The frequency of total CD3^+^, CD4^+^, and CD8^+^ cells were predominantly higher in peritumoral region of low responders compared to high responders. (IHC 20×; 200 µm scale, in set: 40×) (**B**) The induction of sixteen different genes was performed using qPCR following stimulation with aAPC compared to cells cultured in medium alone and normalized to the housekeeping control (18S). A heatmap showing the expression patterns of 16 immune-associated genes in four high responders versus three low responders. Each colored square on the heatmap represents the relative median score for the number of samples with highest expression being red, lowest expression being green, and average expression being black. (**C**) Bar graph displaying the genes in which statistically significant differences were observed in donors with high NKT cell function (n = 4) compared to donors with no detectable NKT cell function (n = 3), as assessed by IFN-γ induction. Each experimental specimen performed in triplicate. Welch T test was performed with *p* ≤ 0.05 considered significant.

We sought to determine if there was a correlation between the peripheral blood and tumor microenvironment. Therefore, we assessed the percentage of tumor-infiltrating lymphocytes (TILs) using CD3, CD4, CD8, and PD-L1 expression within the tumor microenvironment by immunohistochemistry (Figure 4A). The frequency of total TILs, CD3^+^ cells, CD4^+^ cells, and CD8^+^ cells was predominantly higher in peritumoral as well as intratumoral regions of low responders compared to high responders (Supplemental Table S1). No significant difference was observed regarding PD-L1 between these two groups.

We next compared gene profiles of BC patients with high (n = 4) versus low (n = 3) NKT cell function. The induction of sixteen different immune-related genes was assessed using qPCR following stimulation of cells with aAPC or medium alone, and values were normalized to the housekeeping control (18S) (Figure 4B). A clustergram depicting gene expression of candidate genes evaluated by qPCR is shown in Figure 4B. We found that low responders had a significant reduction in the induction of TNF-α (*p* value = 0.03), LAG3 (*p* value = 0.009), and LIGHT (*p* value = 0.007) (Figure 4C). We confirmed differential expression of these target genes in the entire cohort of BC patients (Supplemental Figure S1). Collectively, these data show that in breast cancer patients, circulating NKT cell number may not correlate with function and provide a subset of biomarkers TNF-α, LAG3, and LIGHT, which can be used to assess immune function.

### 2.5. Pretreatment with BC-Derived Conditioned Medium Abrogates NKT Cell Activation

**Figure 5:**
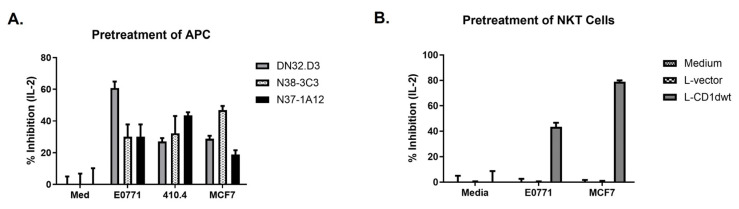
Pretreatment with breast cancer cell supernatants inhibits NKT cell activation. (**A**) LCD1d1wt cells were treated with fresh media or conditioned culture media for 4 h, then washed extensively and cocultured with NKT cell cells. (**B**) NKT cells were pretreated with BC-conditioned media, washed, and cocultured with controls (media, L-vector) or antigen-presenting cells (LCD1dwt). IL-2 was measured, as an indication of NKT cell activation.

We next sought to investigate the mechanisms regulating NKT cell number and function in BC patients. It is known that hyperlipidemia is present in both mammary tissue and sera (8) of BC patients, thus we investigated whether conditioned medium from BC cells inhibited CD1d-mediated NKT cell activation. Tumor cells shed gangliosides and other glycolipids that can inhibit T cell activation, as well as impede the generation of anti-tumor CD8^+^ T cells [24]. NKT cells have been reported to recognize gangliosides in the context of CD1d [25], and breast cancer cells have been shown to overexpress GD3 [26]. We collected conditioned medium, cell-free culture supernatant as previously reported [27,28], from human (MCF-7) and mouse (E0771 and 410.4) breast cancer cell lines, and used the medium to treat CD1d-expressing cells (LCD1dwt). Following treatment, LCD1dwt cells were washed extensively and co-cultured with NKT cells. We found that the ability of the CD1d-expressing cells to induce NKT cell cytokine production was reduced by 40–60% following treatment with the cultured supernatant from the breast cancer cell lines, as shown in Figure 5A. Interestingly, in the reverse experiment, when NKT cells were pretreated with the tumor cell supernatants, their function was also reduced (Figure 5B). These data suggest that breast cancers secrete substances, which can directly and indirectly (by affecting the antigen-presenting cell) inhibit NKT cell function.

**Figure 6:**
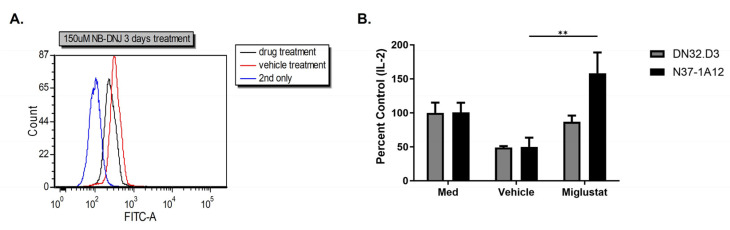
Treatment of BC cells with a ganglioside inhibitor can restore CD1d-mediated NKT cell activation. MCF7 cells were pretreated with 150 μM Miglustat or vehicle (water) for 3 days, washed, and cultured in fresh medium. (**A**) GD3 expression in MCF7 cells. (**B**) LCD1dwt cells were treated with medium (EMEM), vehicle-treated conditioned medium (CM), or Miglustat-treated CM for 4 h, washed, and co-cultured with NKT cells. A two-way ANOVA with Tukey multiple comparison test was performed with *p* ≤ 0.05 considered significant. ** *p* ≤ 0.01.

We have previously investigated the role of the tumor-associated ganglioside GD3 in inhibiting CD1d-dependent NKT cell activation [29]. We have found that both ovarian cancers [29] and breast cancers overexpress GD3 (Figure 6A). Moreover, we have found that GD3 binds with high affinity to CD1d molecules and competes with activating lipid antigen, resulting in a loss of NKT cell activation [29]. Thus, we next investigated whether treatment with a glycosphingolipid inhibitor could restore CD1d-mediated NKT cell activation. As shown in Figure 6B, we found that pretreatment of breast cancer cells with Migulstat (N-butyldeoxynojirimycin), a drug that blocks the synthesis of GD3, restores CD1d-mediated NKT cell activation. Importantly, these data show that ganglioside inhibitors can be used to abrogate the production of immunosuppressive lipids by BC.

**Figure 7:**
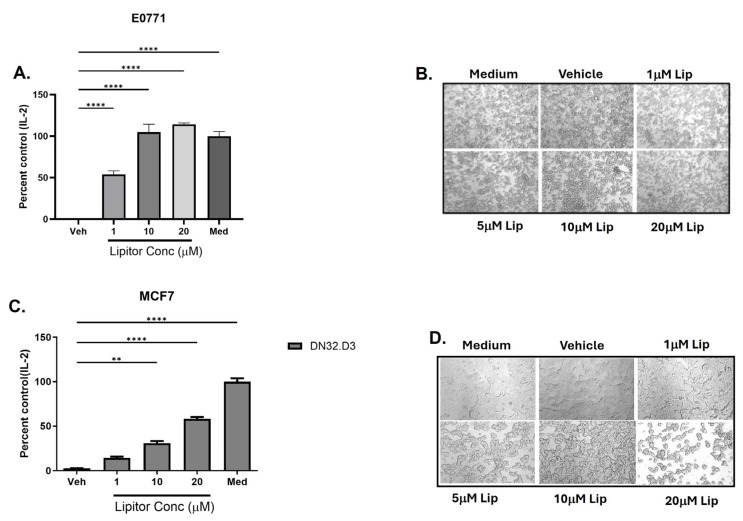
Treatment of MCF7 cells with Lipitor restores NKT cell function. (**A**) Murine E0771 or (**C**) human MCF7 cells were pretreated with Lipitor or vehicle (DMSO) for 3 days, the cells were cultured in fresh medium for two days, and the culture supernatant were used to treat NKT cell hybridomas. The treated NKT cells were cultured with LCD1dwt cells. Treatment with Lipitor inhibits the growth of (**B**) murine E0771 and (**D**) human MCF7 cells. Magnification: 10×. A two-way ANOVA with Tukey multiple comparison test. Med indicates culture media control. ** *p* ≤ 0.01, **** *p* ≤ 0.0001.

During the course of our studies, we found that while both mouse and human BC lines express GD3, pretreatment with GD3 did not directly block NKT cell function (Supplemental Figure S2); rather, it indirectly modulated NKT cell activation by abrogating CD1d-mediated antigen presentation to NKT cells, as previously reported and shown in Figure 6. In order to target BC-mediated direct and indirect suppression of NKT cells, we next examined a different class of inhibitors, specifically statins. Commonly used to treat hyperlipidemia, statins have also been shown to modulate percent NKT cell in non-cancer patients. It has been reported that simvastatin significantly increased NKT cell levels in the peripheral circulation of hyperlipidemic patients as compared to baseline after 6 months of treatment, independent of changes in total or LDL cholesterol [30]. This phenomenon was observed with simvastatin, which inhibits HMG-CoA reductase, but not ezetimibe, which inhibits the intestinal absorption of cholesterol [30]. We found that treating BC cells (MCF7) with the HMG-CoA reductase inhibitor, atoravastatin/Lipitor resulted in a dose-dependent restoration of IL-2 production, a measure of NKT cell function (Figure 7A,C). These data suggest that breast cancer-associated lipids can both directly and indirectly suppress NKT cell activation. Moreover, when we assessed the morphology and proliferation of the treated cells, it was found that treatment with Lipitor resulted in a decrease in cell proliferation (Figure 7B,D).

### 2.6. Treatment with Lipitor Increases NKT Cell Number and Function In Vivo

**Figure 8:**
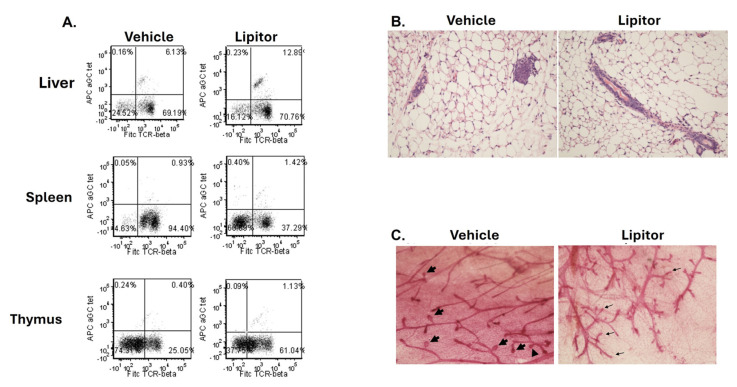
Treatment of Brca-1 mutant mice with Lipitor can increase NKT cell number and reduces mammary epithelial cell growth. Brca-1 mutant mice were treated with Lipitor for 4 weeks. (**A**) The liver, spleen, and thymus were analyzed for % NKT cells by flow cytometry. (**B**) Atypical ductal hyperplasia with formations of micro lumen was observed in vehicle-treated BRCA-1 mutant mice. In contrast, in Lipitor-treated mice, no structural changes in the mammary gland were observed. Magnification, 20×. (**C**) Representative whole mounts of mice treated with vehicle (**left**) or Lipitor (**right**) as described. Thick arrows indicate areas of abnormal dense mammary epithelial cell growth at the end of terminal ducts in vehicle-treated mice (5 out of 6 mice) compared to normal appearing terminal ducts in Lipitor-treated mice (thin arrows) (6 out of 7 mice). Magnification, 40×.

Next, we sought to examine whether treatment with Lipitor could restore NKT cell number and function in vivo. The breast cancer susceptibility gene 1 (*BRCA1*) encodes a tumor suppressor gene that functions, in part, as a caretaker gene in preserving chromosomal stability [31]. Mutations in BRCA1 account for the majority of hereditary breast cancer and predominantly result in truncation of the BRCA1 protein [31,32]. Thus, we sought to investigate whether treatment with a statin would impact NKT cell frequency, and examine the mechanisms underlying mammary epithelial cell growth and differentiation in BRCA1-associated breast cancers using a Brca1 mutant model [33]. As recently reported, when we initially examined the NKT cell population of BRCA-1 mutant mice, we found that the NKT cell population was significantly reduced, compared to wild type C57BL/6 mice, which represents an 82% reduction in the NKT cell population, similar to what was observed in BC patients [34]. However, through additional studies we determined that the loss of the exon 11 mutation did not directly impact NKT cell development [34]. Therefore, by utilizing littermate controls, we investigated whether treatment with Lipitor would restore NKT cell number and function in Brca-1 mutant mice and if it would have an effect on tumor development. Brca-1 mutant mice were treated with Lipitor for five days a week for four weeks. Importantly, it was found that treatment with Lipitor not only restored NKT cell % (Figure 8A), but treatment also resulted in a decrease in hyperplastic lesions in BRCA mice (Figure 8B,C).

**Figure 9:**
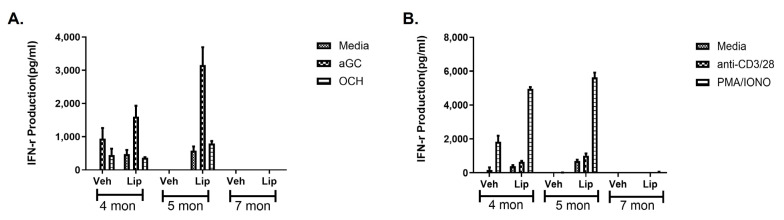
Early treatment of Brca-1 mutant mice with Lipitor can restore both NKT and T cell function. Three, four, and six-month old mice were treated with Lipitor for 4 weeks. (**A**) Splenocytes were cultured in medium or the indicated NKT cell agonists or (**B**) stimulated with anti-CD3/CD28 beads or PMA/ionomycin for 48 h. Culture supernatants were harvested and standard sandwich ELISA was used to measure IFN-γ production. A two-way ANOVA with Tukey multiple comparison test was conducted and all of the four- and five-month treatment groups were significantly different compared to the media control (*p* ≤ 0.001).

Thus, we investigated whether treatment with Lipitor would restore NKT and total cell function in Brca-1 mutant mice. Time course studies were performed in which Brca-1 mutant mice were treated with Lipitor for four weeks, and splenocytes were harvested and stimulated with a-GalCer and OCH to assess NKT cell responses (Figure 9A) or anti-CD3/CD28 microbeads or PMA/ionomycin (Figure 9B) to assess general immune responsiveness. Strikingly, we found that treatment enhanced NKT cell and total T cell responses in younger mice compared to older BRCA mutant mice.

## 3. Discussion

Tumor resistance and immune suppression remain challenging obstacles to the efficacy of immunotherapies [35,36,37]. We report here the feasibility of measuring circulating NKT cell function as a correlation of immune function in BC patients. To the best of our knowledge, this is the first study to demonstrate a discrepancy between NKT cell number and function in BC.

NKT cells are a potent immunoregulatory T cell subpopulation involved in promoting anti-tumor immunity [38]. NKT cells are known to bridge the innate and adaptive immune systems, which enhances their protective immune responses [39]. NKT cells have direct and indirect anti-tumor functions, including the ability to activate both innate NK cell and adaptive T cell-mediated anti-tumor immunity [40,41,42]. Therefore, NKT cells play an important role in immune surveillance. In support of this concept, clinical trials using NKT cell-based therapy have demonstrated prolonged median survival time in lung cancer patients [43,44] and achieved stable disease or partial responses in head and neck cancer patients [45]. Of note, no significant toxic side effects were observed.

However, several studies have reported that NKT cell numbers are significantly reduced in cancer patients [18,46,47]. Thus, the low frequencies of NKT cells in peripheral blood have limited their application for clinical approaches. Despite reduced NKT cell counts in cancer patients, their strong anti-tumor potential has resulted in several trials focused on activating NKT cells [48,49,50]. A phase 1 clinical trial was performed using in vitro expanded, autologous iNKT cells in stage IIIB-IV melanoma [51]. Taken together with recent studies showing that NKT cell-based therapy can be combined with chemotherapy to reduce tumor burden and enhance protection against tumor recurrence, we sought to develop an assay that could be used to measure NKT cell function in cancer patients. NKT cell function is routinely evaluated by traditional assays such as ELISA and ELISPOT. However, these methods require a large volume of blood or in vitro expansion, and can display limited sensitivity due to the low number of circulating NKT cells. Herein, we describe a method that can be used to rapidly measure NKT cell function, as assessed by the induction of IFN-γ, TNF-α, LIGHT, and LAG-3.

Robust evidence supports the prognostic and therapy-predictive roles of immune gene signatures in breast cancer. However, the functional relevance of these biomarkers in immunotherapy remains unclear. Research aimed at dissecting the immunological features of tumors could aid in our understanding of the inherent differences in patient immune responses, as well as underlying mechanisms that regulate anti-tumor immunity and its therapeutic employment in breast cancer. The identification of peripheral biomarkers that indicate the level of tumoral immune response could allow us to develop less invasive diagnostics tools for immune responsiveness. However, it is currently unclear whether the predictive information identified by peripheral blood biomarkers correlates with tumor immune signatures. Therefore, we investigated the prognostic significance of BC patients with functional NKT cells (high IFN-γ responders) and nonfunctional NKT cells (low IFN-γ responders) (Figure 4B). Following further characterization of these groups, it was found that low responders had a significant reduction in the induction of TNF-α, LAG3, and LIGHT. LAG-3 (CD223) is a cell surface molecule expressed on activated T cells [52], NK cells [53], B cells [54], and plasmacytoid dendritic cells. A trial combined IMP321 (LAG-3Ig) with conventional chemotherapy in 33 women with metastatic breast cancer and observed increased immune responses and anti-tumor activity [55]. LIGHT is a ligand for the TNF superfamily 14 (TNFRSF14) [56]. This protein has been shown to stimulate the proliferation of T cells and trigger apoptosis of various tumor cells [57]. In addition, TNFRSF14 is found in tertiary lymphoid structures (TLS) with features that resemble the characteristics of lymph nodes in melanoma [57]. Moreover, human NK-cell activation can lead to the induction of TNFRSF14 expression [58]. However, further characterization of these immune biomarkers in BC is clearly warranted.

Studies that seek to disentangle unique and overlapping genes comprising numerous published signatures, and in treatment-specific contexts, are needed to optimize clinical implementation strategies. Assessing factors produced by tumor-infiltrating lymphocytes (TILs) as well as stromal cells within the tumor microenvironment will be important in developing a predictive biomarker profile [59]. TILs have been suggested to be a manifestation of host immune reactions to cancer cells [60]. Analysis of baseline TILs has shown prognostic value in large clinical series [61,62,63]. Although many groups are currently evaluating TIL, there is no standardized system for diagnostic applications. A previous study on 30 breast cancer patients demonstrated the positive correlation between T cells in the periphery and the tumor environment [64]. To investigate whether the immunological responses in the peripheral blood reflect the composition of immune cells at the tumor site, we investigated the percentage of TILs and PD-L1 expression within the tumor microenvironment by immunohistochemistry (IHC) (Figure 4A). IHC analysis of TILs showed slightly higher densities of TILs in peri- and intratumoral areas of low responders compared to high IFN-γ responders. We did not observe any significant difference in PD-L1 between these two groups. Previously, Platzer et al. reported differences in TILs and peripheral lymphocytes in breast cancer patients [65]. In future studies, we will use laser capture microdissection to assess TILs by qPCR rather than IHC. This may open the way for standardized reporting of tumor immunological parameters in clinical studies. Research aimed at dissecting the phenotypic and biological attributes of tumors that statistically modify the immune signature–patient outcome associations (such as proliferation and subtype) could shed valuable light on immunogenic differences among patient populations, as well as on the underlying mechanisms that regulate antitumor immunity and its therapeutic manipulation in breast cancer. Furthermore, it is unknown whether the prognostic and predictive information captured by tumor immune signatures can be predicted by the assessment of peripheral blood markers. The identification of peripheral parameters that indicate the level of intratumoral immune response could enable the development of less invasive diagnostics for immune responsiveness.

Our previous studies on ovarian cancer demonstrated the role of the immunosuppressive lipid GD3 on CD1d-mediated NKT cell activation. Here, we found that BC also expresses GD3 and that inhibition of GD3 restored CD1d-mediated NKT cell responses. However, we hypothesize that alterations in metabolism result in changes in the lipid repertoire and that BC expresses other immunosuppressive lipids, in addition to GD3. Our studies focused on how metabolic alterations during malignant transformation affect CD1d-dependent NKT cell activation were initiated by our studies focused on hypoxia-inducible factor (HIF)-1α [66]. As HIF plays a key role in metabolism, we hypothesized that induction of HIF-1α or alterations in glycolytic metabolism may be the mechanism by which cancers regulate NKT cell responses [66], by either presenting activating or inhibitory lipid antigen in the context of CD1d. Herein, we observed an increase in NKT cell responses when we treated BC cell lines and *Brca-1* mutant mice with Lipitor. Notably, in our in vivo studies, we sought to use a physiologically relevant concentration of Lipitor. Patients are generally prescribed 10–80 mg/day, which results in circulating plasma levels of 0.9–6 ng/mL, thus our in vitro studies (20 μM) were a little high. In patients taking 80 mg/day for 6 weeks the circulating levels were 6 ± 8.2 ng/mL, based on the literature, thus we weighed the mice daily and used a physiologic concentration. Importantly, we found that patients prescribed statins had higher NKT cell function, compared to those who were not. In the future, we will investigate the mechanisms by which treatment with drugs that modulate metabolism enhance NKT cell responses. We will also work to identify novel targets that can be used as biomarkers. In addition, potential candidates will be investigated further in order to help develop new therapeutics.

## 4. Materials and Methods

### 4.1. Study Design

Peripheral blood was collected from newly diagnosed breast cancer patients at the University of Maryland School of Medicine (UMSOM) prior to treatment. The Institutional Review Board of the UMSOM approved this study. Written informed consent was obtained from all breast cancer patients before participation in this study. Healthy donor blood samples were purchased from commercial vendors such as Biological Specialty Corporation, now BioIVT (Westbury, NY, USA).

### 4.2. Human Peripheral Blood Mononuclear Cell (PBMC) Isolation

Breast cancer patient PBMCs were isolated with BD Vacutainer CPT Tubes for Molecular Diagnostics (20-959-51D; Fisher Scientific, Suwanee, GA, USA). PBMCs were isolated from healthy donor buffy coats by Ficoll-Hypaque (Amersham Pharmacia Biotek, Uppsala, Sweden) using SepMate-50 tubes (Stemcell technologies, Vancouver, BC, Canada), according to the manufacturer’s instructions.

### 4.3. Generation of Artificial Antigen-Presenting Cells (aAPCs)

The CD1d-aAPCs were prepared as previously reported [67]. Briefly, to conjugate hCD1d-Ig dimer molecules to beads, hCD1d-Ig (BD Pharmingen, Franklin Lakes, NJ, USA) and anti-CD28 mAb (Biolegend, San Diego, CA, USA) was added to M-450 Dynabeads (ThermoFisher, Waltham, MA USA). The beads were subsequently washed and loaded with α-Galactosylceramide (Diagnocine, Hackensack, NJ, USA).

### 4.4. Stimulation of PBMCs

PBMCs were simulated for 4 h at 37 °C with anti-CD3/CD28 mAb-coated microbeads and α-GalCer-loaded aAPC. Cell culture medium was used as a negative control and PMA (50 ng/mL) and ionomycin (1 μM) was used as a positive control.

### 4.5. Cell Lines

Human breast cancer cells, MCF-7, and mouse breast cancer cell lines (410.4 and E0771) were cultured in McCoy’s 5a Modified Medium supplemented with 10% fetal bovine serum and penicillin/streptomycin.

Murine L cells transfected with vector alone or the WT *cd1d1* cDNA in pcDNA3.1-neo (Invitrogen, Waltham, MA, USA) (LCD1dwt) were kindly provided by R.R. Brutkiewicz (Indiana University School of Medicine, Indianapolis, IN, USA) [68], and were cultured in DMEM media, supplemented with 2 mM L-glutamine, 10% FBS, 500 μg/mL G418, and penicillin/streptomycin. The cell lines used have been tested and authenticated routinely by staining for stable cell surface expression of CD1d, compared to isotype control staining, and also compared to cells stably transfected with the empty control vector.

The Va14^+^ NKT cell hybridoma cell lines DN32.D3 [69,70], N38-2C12, N38-2H4, N38-3C3, and the CD1d1-specific NKT cell hybridoma N37-1A12 (Va5^+^), have all been described [71] and were cultured in IMDM medium supplemented with 5% FBS and 2 mM L-glutamine. The NKT cells are tested for specificity to CD1d in each experiment via functional T cell assay. Cells were imaged under an Olympus CK2 light microscope from Olympus Corporation (Tokyo, Japan) using a 10× objective. Images were captured using an Olympus Pen E-PL1 digital camera from Olympus Corporation (Tokyo, Japan).

### 4.6. Treatment of Cells with Condition Medium

Conditioned media was collected from cancer cell lines cultured in T-75 flasks after 72–96 h. The media was centrifuged to obtain cell-free supernatants. APCs were treated with the clarified supernatants (conditioned media) for 4–6 h at 37 °C, unless otherwise indicated. The APCs were subsequently washed extensively with PBS and cocultured with NKT hybridomas (0.5–1 × 10^5^) for 20 to 24 h at 37 °C. IL-2 and IFN-γ cytokine release was measured as an indication of NKT cell activation and was assessed by standard sandwich ELISA.

### 4.7. ELISAs

IFN-γ levels following stimulation of PBMC was determined by standard sandwich ELISA (eBioscience, San Diego, CA, USA). Cytokine release from mouse cells was measured as an indication of murine NKT/T cell activation and was measured by standard sandwich ELISA (Biolegend).

### 4.8. RNA Isolation

RNeasy Plus mini kit from Qiagen was used to extract RNA. The quality of the total RNA was verified by 2% agarose gel electrophoresis. RNA was reverse transcribed using the iScript cDNA Synthesis Kit (Biorad, Hercules, CA, USA) according to manufacturer’s instruction.

### 4.9. Real-Time Quantitative PCR (qPCR)

qPCR was performed using primers specific for 18S as an internal control, and nineteen different primers, purchased from Applied Biosystems TaqMan Assays (ThermoFisher, Waltham, MA USA). The total volume of the reaction mix was 20 μL and consisted of 10 μL master mix, 1 μL primer mix, 7 μL H_2_O, and 2 μL cDNA. Cycling was performed at 50 °C for 2 min, 95 °C for 10 min, 40 cycles at 95 °C for 15 s, and 40 cycles at 60 °C for 1 min. The same thermal profile conditions were used for all primer sets. For investigation of the robustness of this method, the performance of the qPCR was additionally assayed with a Brilliant III ultra-fast QPCR master mix kit (Agilent Technologies, Santa Clara, CA, USA) using AriaMx Real-Time PCR system (Agilent Technologies, USA) per the manufacturer’s instructions. The CT values were collected, and the fold increase calculated as follows: n-fold increase in IFN-γ mRNA = 2[−(CT_sample_ − CT 18S rRNA) − (CT_empty beads_ − CT 18S rRNA)] where CT is the threshold cycle.

### 4.10. Antibodies and Flow Cytometry

In order to detect the number of total T cells and NKT cells we conducted flow cytometry. Human antibodies were used as follows: anti-TCR Vα24-Jα18 (clone 6B11, Biolegend), anti-CD3 (clone UCHT1, BD Biosciences, Franklin Lakes, NJ, USA), anti-CD3/CD4/CD8 (Biolegend). Data were collected on an LSR II from BD Biosciences and analyzed using FCS Express De Novo Software (Version 5, Los Angeles, CA, USA).

### 4.11. Mouse Studies

Post-pubertal Brca1 conditional knockout mice (used at 3, 4, 6 months of age) with two floxed Brca1 alleles (Brca1f/f) carrying the mouse mammary tumor virus (MMTV)-Cre recombinase gene (Brca1f/f; MMTV-Cre) were maintained on a C57Bl/6 genetic background [33]. Mice were maintained in temperature-controlled and light-controlled conditions in the University of Maryland, Baltimore animal facility. All mice were maintained in accordance with institutional guidelines approved by the University of Maryland, Baltimore Animal Care and Use Committee. The presence or absence of the floxed Brca1 alleles, of wild-type Brca1 alleles, and of the MMTV-Cre was identified using polymerase chain reaction (PCR) on tail DNA as described previously [33]. BRCA-1 mutant mice were treated once a day (5×/week) for 4 weeks with Lipitor (10 mg/kg) or vehicle (phosphate-buffered saline) intraperitoneally (i.p.). Liptor (Atorvastin). Mice were weighed daily. Fourth mammary glands were surgically removed at necropsy and processed for a whole mount analysis or formalin fixed for histology.

### 4.12. Whole Mount and Histology

One #4 mammary gland from each animal was dissected and spread on a glass slide at the time of necropsy for whole mount analyses as previously described [72,73]. The other #4 mammary gland from each mouse was fixed in 10% buffered formalin (Fisher Scientific, Pittsburgh, PA, USA) overnight at 4 °C and embedded in paraffin using standard techniques. Five-micron sections were cut for hematoxylin and eosin (H&E) staining. Digital photographs were taken using a Nikon 50i Upright Microscope System with a high resolution, 5 Megapixel Color Digital Camera system (Nikon Instruments Inc., Melville, NY, USA).

### 4.13. Statistical Analyses

For all bar graphs, bars represent means ± SEM. Statistical analyses were performed using Prism 10.02.03 software (GraphPad). * *p* ≤ 0.05, ** *p* ≤ 0.01, *** *p* ≤ 0.001, **** *p* ≤ 0.0001.

## 5. Conclusions

While cancer immunotherapy has revolutionized the field and ushered in a new era of treatment options, it has had limited success in breast cancer. In this study, we sought to determine which breast cancer patients would benefit from NKT cell-based therapy and to determine the mechanism by which breast cancers suppressed NKT cell responses. Overall, we found that NKT cells were reduced in breast cancer patients, compared to healthy donors; however, many patients had functional NKT cells. When we compared BC patients with highly functional NKT cells, termed responders, to those with little to no IFN-γ induction after stimulation of NKT cells, there was no significant difference in NKT cell number between the groups, suggesting that breast cancer-associated NKT cells were functionally impaired. When we examined immunosuppressive lipid shedding by breast cancers, we found that treatment of breast cancer cell lines and Brca-1 mutant mice with Lipitor restored anti-tumor immune responses and decreased mammary hyperplasia in vivo. Our studies have several limitations; for example, the number of patients in our study was low and the animal model represented a specific type of breast cancer. Therefore, in future studies we would like to conduct a more comprehensive study and compare circulating immune cells (blood) to local immune responses (within the tumor microenvironment) to determine whether the repurposed drugs are having a local effect as well as systemic effect on the immune system. In addition, we would like to test our therapeutic strategy of using statins to modulate anti-tumor immunity in different models of breast cancer.

## Figures and Tables

**Table 1:** Demographic and clinical–pathologic characteristics of breast cancer patients according to NKT cells number and function.

Characteristics	Number of Patients	Avg. NKT Cell %	NKT Cell Function
**Age (years)**			
**≤50**	15	0.044 ± 0.04	4.40 ± 10.7
**>50**	15	0.038 ± 0.05	3.61 ± 7.9
**Race**			
**White**	13	0.046 ± 0.05	1.47 ± 1.04
**≤50**	7	0.031 ± 0.03	1.03 ± 0.6
**>50**	6	0.065 ± 0.06	1.98 ± 1.2
**African American**	14	0.032 ± 0.04	6.77 ± 13.2
**≤50**	5	0.056 ± 0.06	10.52 ± 18.2
**>50**	9	0.02 ± 0.02	4.7 ± 10.2
**Asian**	3	0.056 ± 0.01	2.08 ± 0.8
**≤50**	3	0.056 ± 0.01	2.08 ± 0.8
**>50**	0	0	0
**Histologic type**			
**Ductal**	24	0.038	4.71
**Lobular**	3	0.076	1.26
**Tubular**	1	0.03	1.43
**Colloid**	1	0.05	0.5
**Not indicated**	1	0	1.55
**ER+**	21	0.037	3.4
**PR+**	17	0.035	3.85
**ER+/HER2+**	2	0.02	1.85
**TNBC**	8	0.05	7.72
**Staging**			
**I**	11	0.025	1.06
**II**	12	0.058	5.67
**III**	6	0.043	6.5
**Tumor grade**			
**1**	9	0.015	1.41
**2**	11	0.061	5.39
**3**	9	0.046	5.18

Abbreviations: ER, estrogen receptor; PR, progesterone receptor; HER2, human epidermal growth factor receptor; TNBC, triple-negative breast cancer.

**Table 2:** Characteristics of patients with low NKT cell function compared to patients with high NKT cell function.

**Low** **Responder** **ID**	**Age at Dx**	**Race**	**BMI**	**Pathology**	**Classification**	**Grade**	**Stage**	**NKT Cells %**	**NKT Cell Function**	**T Cell Function**
**3**	63	B	37.3	Invasive colloid	ER+/PR+	1	I	0.05	0.5	0
**16**	49	W	21.2	Invasive ductal carcinoma	ER+/PR+	1	I	0	0.1	0.7
**29**	49	B	27.6	Invasive ductal carcinoma	ER+/PR+	2	I	0	0.5	3.7
**30**	47	W	23.7	Invasive ductal carcinoma	ER+/PR+	3	I	0.06	0.5	8.6
**33**	43	W	36.5	Invasive ductal carcinoma	TNBC	3	IIA	0.08	0.5	6.2
**High Responder** **ID**	**Age at Dx**	**Race**	**BMI**	**Pathology**	**Classification**	**Grade**	**Stage**	**NKT Cell %**	**NKT Cell Function**	**T Cell Function**
**6**	43	AS	21.0	Invasive ductal carcinoma	ER+/PR+	2	II	0.07	3.1	23.4
**11**	60	W	27.6	Invasive ductal carcinoma	TNBC	2	II	0.2	4	2.7
**13**	47	B	29.2	Invasive ductal carcinoma	ER+/PR+	2	II	0.1	43	3
**18**	54	B	70.3	Invasive ductal carcinoma	TNBC	3	IIIB	0.02	32	8.6
**27**	49	B	39.6	Invasive ductal carcinoma	ER+/PR+	1	II	0	4.5	114

Abbreviations: ER, estrogen receptor; PR, progesterone receptor; TNBC, triple–negative breast cancer.

## Data Availability

Raw data are available upon request.

## Supplementary Materials

The following supporting information can be downloaded at: https://www.mdpi.com/article/10.3390/ijms252212309/s1.
