# Supplementary material for: Identification of Functional Immune Biomarkers in Breast Cancer Patients
Source: Int J Mol Sci. 2024 Nov 16;25(22):12309. doi: 10.3390/ijms252212309 (PMC11595306; doi:10.3390/ijms252212309)
Supplement: Supplementary file 1 [file ijms-25-12309-s001.zip › ijms-3246320-supplementary.pptx]

## Slide 1
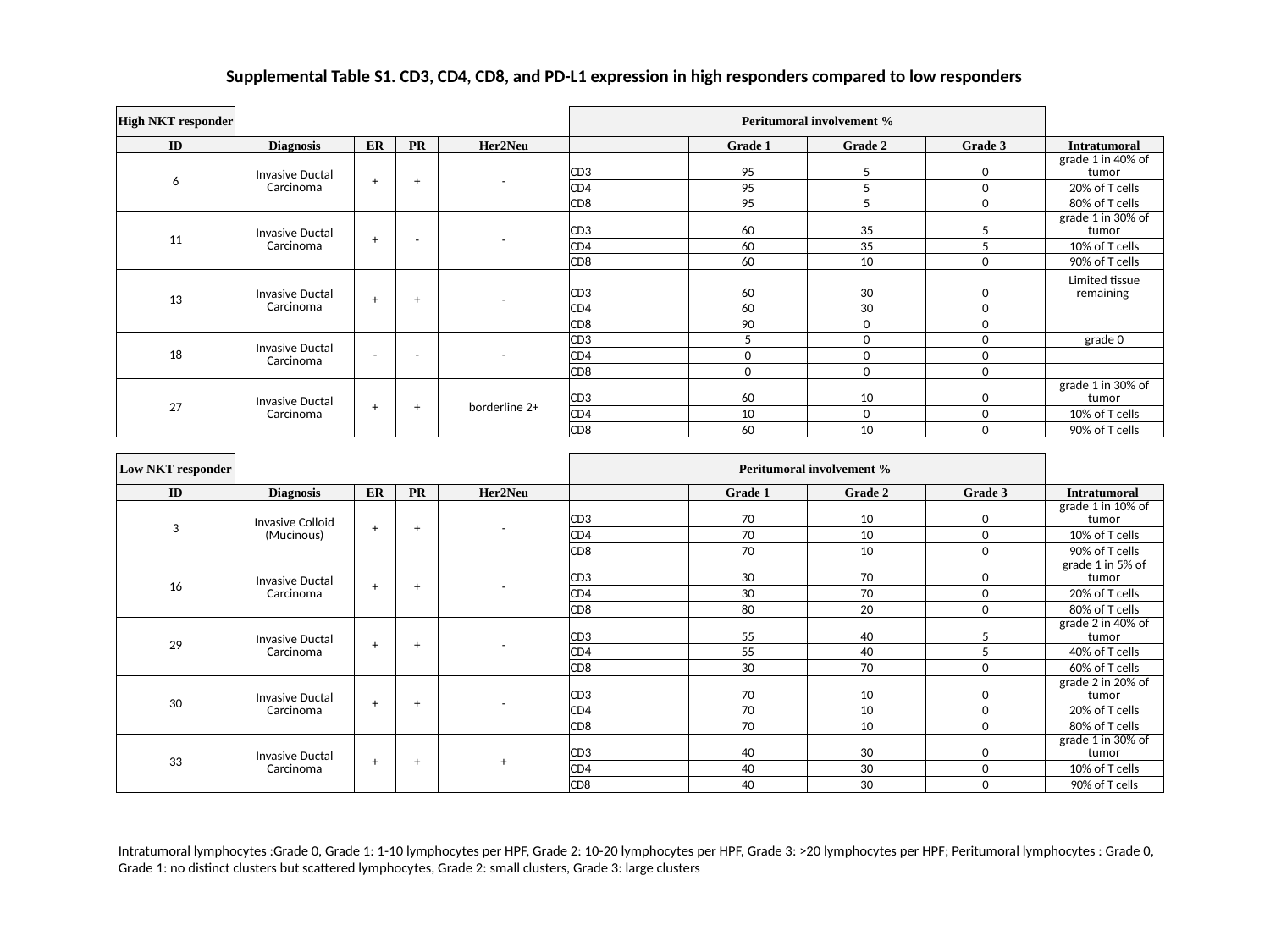

Supplemental Table S1. CD3, CD4, CD8, and PD-L1 expression in high responders compared to low responders
| High NKT responder | | | | | Peritumoral involvement % | | | | |
| --- | --- | --- | --- | --- | --- | --- | --- | --- | --- |
| ID | Diagnosis | ER | PR | Her2Neu | | Grade 1 | Grade 2 | Grade 3 | Intratumoral |
| 6 | Invasive Ductal Carcinoma | + | + | - | CD3 | 95 | 5 | 0 | grade 1 in 40% of tumor |
| | | | | | CD4 | 95 | 5 | 0 | 20% of T cells |
| | | | | | CD8 | 95 | 5 | 0 | 80% of T cells |
| 11 | Invasive Ductal Carcinoma | + | - | - | CD3 | 60 | 35 | 5 | grade 1 in 30% of tumor |
| | | | | | CD4 | 60 | 35 | 5 | 10% of T cells |
| | | | | | CD8 | 60 | 10 | 0 | 90% of T cells |
| 13 | Invasive Ductal Carcinoma | + | + | - | CD3 | 60 | 30 | 0 | Limited tissue remaining |
| | | | | | CD4 | 60 | 30 | 0 | |
| | | | | | CD8 | 90 | 0 | 0 | |
| 18 | Invasive Ductal Carcinoma | - | - | - | CD3 | 5 | 0 | 0 | grade 0 |
| | | | | | CD4 | 0 | 0 | 0 | |
| | | | | | CD8 | 0 | 0 | 0 | |
| 27 | Invasive Ductal Carcinoma | + | + | borderline 2+ | CD3 | 60 | 10 | 0 | grade 1 in 30% of tumor |
| | | | | | CD4 | 10 | 0 | 0 | 10% of T cells |
| | | | | | CD8 | 60 | 10 | 0 | 90% of T cells |
| | | | | | | | | | |
| Low NKT responder | | | | | Peritumoral involvement % | | | | |
| ID | Diagnosis | ER | PR | Her2Neu | | Grade 1 | Grade 2 | Grade 3 | Intratumoral |
| 3 | Invasive Colloid (Mucinous) | + | + | - | CD3 | 70 | 10 | 0 | grade 1 in 10% of tumor |
| | | | | | CD4 | 70 | 10 | 0 | 10% of T cells |
| | | | | | CD8 | 70 | 10 | 0 | 90% of T cells |
| 16 | Invasive Ductal Carcinoma | + | + | - | CD3 | 30 | 70 | 0 | grade 1 in 5% of tumor |
| | | | | | CD4 | 30 | 70 | 0 | 20% of T cells |
| | | | | | CD8 | 80 | 20 | 0 | 80% of T cells |
| 29 | Invasive Ductal Carcinoma | + | + | - | CD3 | 55 | 40 | 5 | grade 2 in 40% of tumor |
| | | | | | CD4 | 55 | 40 | 5 | 40% of T cells |
| | | | | | CD8 | 30 | 70 | 0 | 60% of T cells |
| 30 | Invasive Ductal Carcinoma | + | + | - | CD3 | 70 | 10 | 0 | grade 2 in 20% of tumor |
| | | | | | CD4 | 70 | 10 | 0 | 20% of T cells |
| | | | | | CD8 | 70 | 10 | 0 | 80% of T cells |
| 33 | Invasive Ductal Carcinoma | + | + | + | CD3 | 40 | 30 | 0 | grade 1 in 30% of tumor |
| | | | | | CD4 | 40 | 30 | 0 | 10% of T cells |
| | | | | | CD8 | 40 | 30 | 0 | 90% of T cells |
Intratumoral lymphocytes :Grade 0, Grade 1: 1-10 lymphocytes per HPF, Grade 2: 10-20 lymphocytes per HPF, Grade 3: >20 lymphocytes per HPF; Peritumoral lymphocytes : Grade 0, Grade 1: no distinct clusters but scattered lymphocytes, Grade 2: small clusters, Grade 3: large clusters

## Slide 2
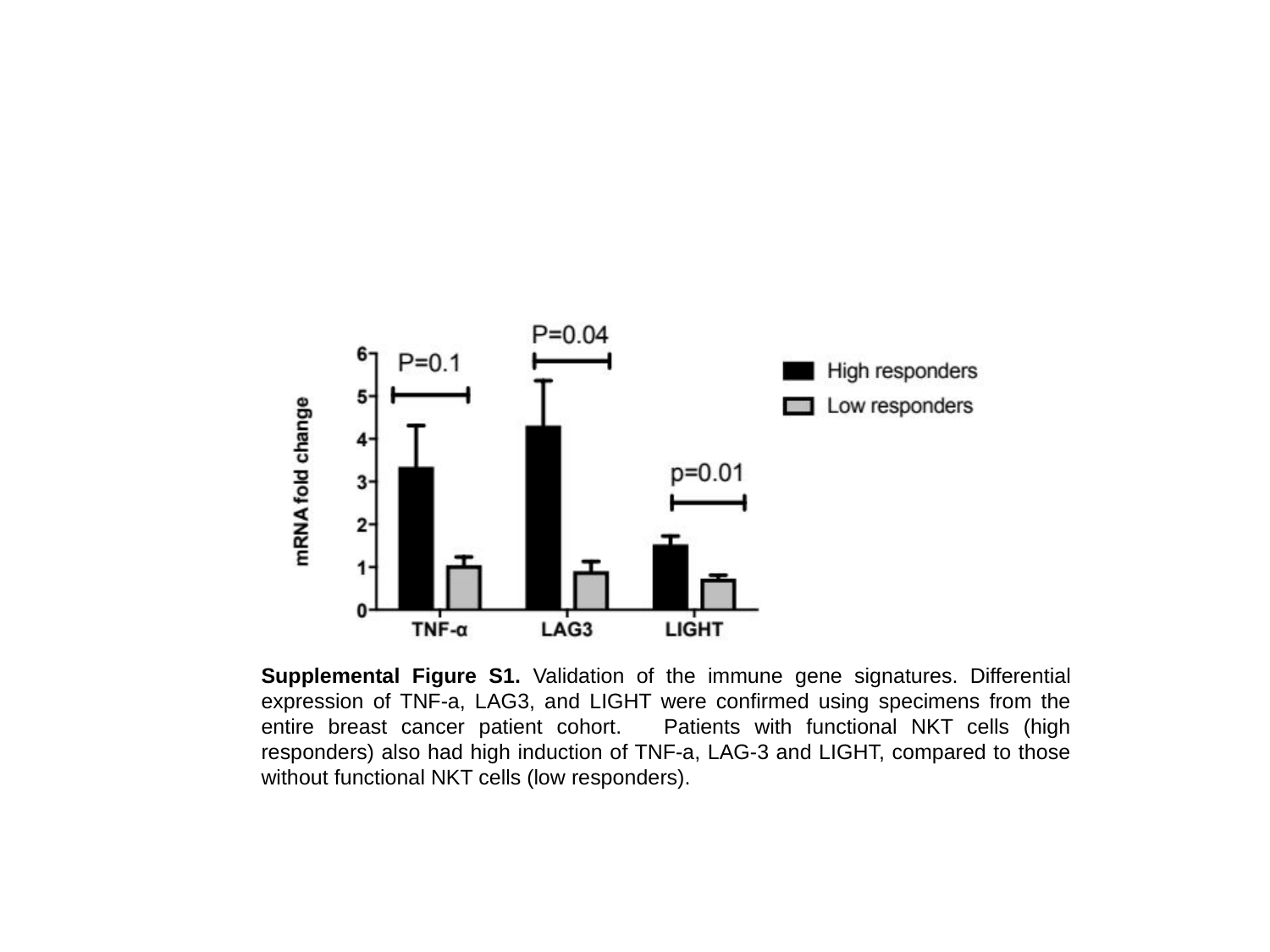

Supplemental Figure S1. Validation of the immune gene signatures. Differential expression of TNF-a, LAG3, and LIGHT were confirmed using specimens from the entire breast cancer patient cohort. Patients with functional NKT cells (high responders) also had high induction of TNF-a, LAG-3 and LIGHT, compared to those without functional NKT cells (low responders).

## Slide 3
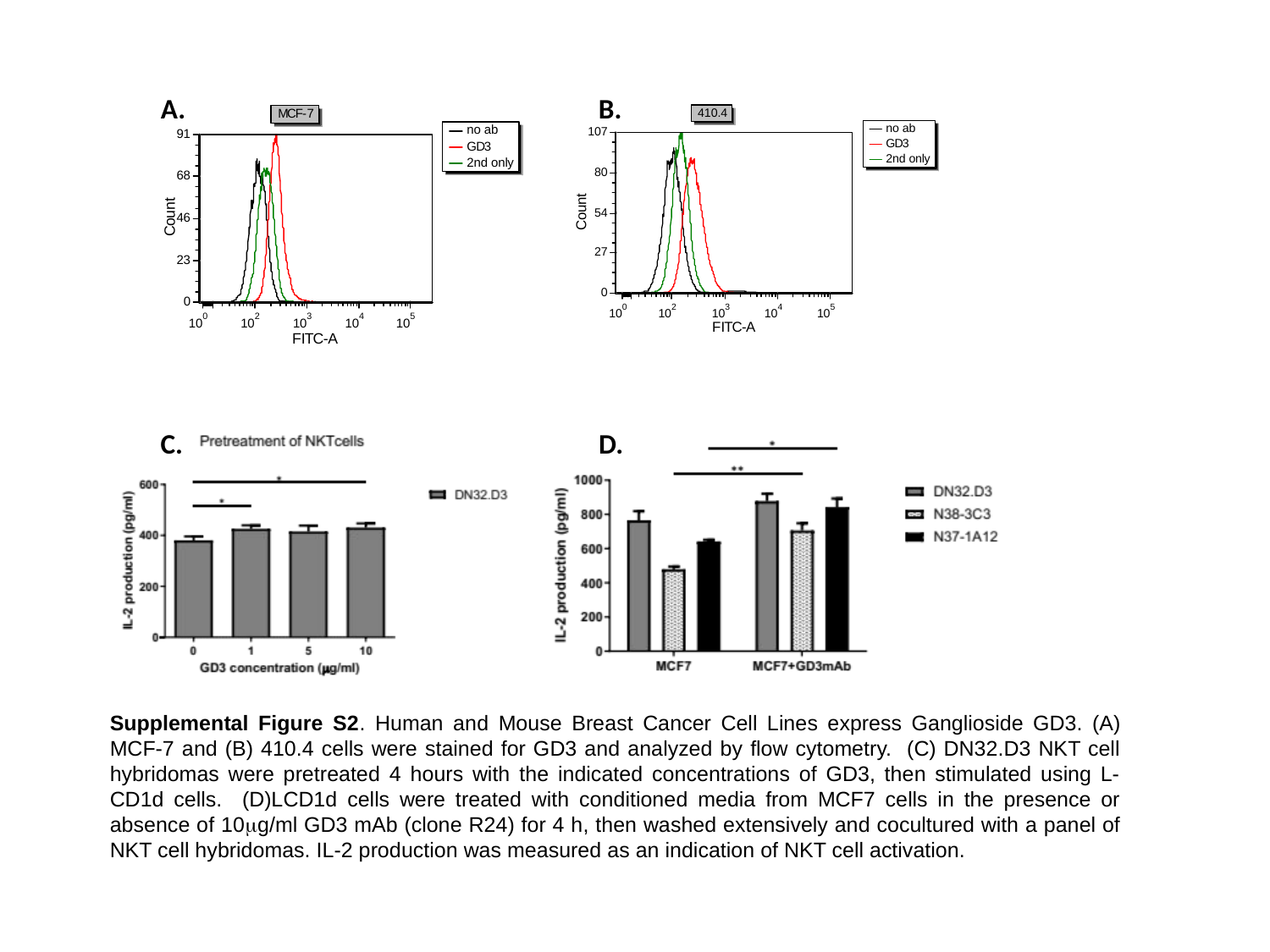

A.
B.
C.
D.
Supplemental Figure S2. Human and Mouse Breast Cancer Cell Lines express Ganglioside GD3. (A) MCF-7 and (B) 410.4 cells were stained for GD3 and analyzed by flow cytometry. (C) DN32.D3 NKT cell hybridomas were pretreated 4 hours with the indicated concentrations of GD3, then stimulated using L-CD1d cells. (D)LCD1d cells were treated with conditioned media from MCF7 cells in the presence or absence of 10mg/ml GD3 mAb (clone R24) for 4 h, then washed extensively and cocultured with a panel of NKT cell hybridomas. IL-2 production was measured as an indication of NKT cell activation.
